# Temporal trends and transmission dynamics of pre-treatment HIV-1 drug resistance within and between risk groups in Kenya, 1986–2020

**DOI:** 10.1093/jac/dkad375

**Published:** 2023-12-13

**Authors:** George M Nduva, Frederick Otieno, Joshua Kimani, Yiakon Sein, Dawit A Arimide, Lyle R Mckinnon, Francois Cholette, Morris K Lawrence, Maxwell Majiwa, Moses Masika, Gaudensia Mutua, Omu Anzala, Susan M Graham, Larry Gelmon, Matt A Price, Adrian D Smith, Robert C Bailey, Patrik Medstrand, Eduard J Sanders, Joakim Esbjörnsson, Amin S Hassan

**Affiliations:** Department of Translational Medicine, Lund University, Lund, Sweden; Department of HIV/STI, KEMRI/Wellcome Trust Research Programme, PO Box 230-80108 Kilifi, Kenya; Nyanza Reproductive Health Society, Kisumu, Kenya; Department of Medical Microbiology, University of Nairobi, Nairobi, Kenya; Department of Medical Microbiology and Infectious Diseases, University of Manitoba, Winnipeg, Canada; Department of HIV/STI, KEMRI/Wellcome Trust Research Programme, PO Box 230-80108 Kilifi, Kenya; Department of Translational Medicine, Lund University, Lund, Sweden; Department of Medical Microbiology, University of Nairobi, Nairobi, Kenya; Department of Medical Microbiology and Infectious Diseases, University of Manitoba, Winnipeg, Canada; Centre for the AIDS Programme of Research in South Africa (CAPRISA), Durban, South Africa; Department of Medical Microbiology and Infectious Diseases, University of Manitoba, Winnipeg, Canada; National Microbiology Laboratory at the JC Wilt Infectious Diseases Research Centre, Public Health Agency of Canada, Winnipeg, Canada; Department of Biochemistry and Biotechnology, Pwani University, Kilifi, Kenya; Kenya Medical Research Institute/Centre for Global Health Research, Kisumu, Kenya; KAVI Institute of Clinical Research, University of Nairobi, Nairobi, Kenya; KAVI Institute of Clinical Research, University of Nairobi, Nairobi, Kenya; KAVI Institute of Clinical Research, University of Nairobi, Nairobi, Kenya; Department of HIV/STI, KEMRI/Wellcome Trust Research Programme, PO Box 230-80108 Kilifi, Kenya; Department of Medicine, Global Health and Epidemiology, University of Washington, Seattle, USA; Department of Medical Microbiology, University of Nairobi, Nairobi, Kenya; Department of Medical Microbiology and Infectious Diseases, University of Manitoba, Winnipeg, Canada; IAVI, NewYork, USA; Department of Epidemiology and Biostatistics, University of California San Francisco, San Francisco, CA, USA; Nuffield Department of Medicine, University of Oxford, Oxford, UK; Nyanza Reproductive Health Society, Kisumu, Kenya; Division of Epidemiology & Biostatistics, University of Illinois at Chicago, Chicago, IL, USA; Department of Translational Medicine, Lund University, Lund, Sweden; Department of HIV/STI, KEMRI/Wellcome Trust Research Programme, PO Box 230-80108 Kilifi, Kenya; Nuffield Department of Medicine, University of Oxford, Oxford, UK; Department of Translational Medicine, Lund University, Lund, Sweden; Nuffield Department of Medicine, University of Oxford, Oxford, UK; Department of Translational Medicine, Lund University, Lund, Sweden; Department of HIV/STI, KEMRI/Wellcome Trust Research Programme, PO Box 230-80108 Kilifi, Kenya

## Abstract

**Background:**

Evidence on the distribution of pre-treatment HIV-1 drug resistance (HIVDR) among risk groups is limited in Africa. We assessed the prevalence, trends and transmission dynamics of pre-treatment HIVDR within and between MSM, people who inject drugs (PWID), female sex workers (FSWs), heterosexuals (HETs) and perinatally infected children in Kenya.

**Methods:**

HIV-1 partial *pol* sequences from antiretroviral-naive individuals collected from multiple sources between 1986 and 2020 were used. Pre-treatment reverse transcriptase inhibitor (RTI), PI and integrase inhibitor (INSTI) mutations were assessed using the Stanford HIVDR database. Phylogenetic methods were used to determine and date transmission clusters.

**Results:**

Of 3567 sequences analysed, 550 (15.4%, 95% CI: 14.2–16.6) had at least one pre-treatment HIVDR mutation, which was most prevalent amongst children (41.3%), followed by PWID (31.0%), MSM (19.9%), FSWs (15.1%) and HETs (13.9%). Overall, pre-treatment HIVDR increased consistently, from 6.9% (before 2005) to 24.2% (2016–20). Among HETs, pre-treatment HIVDR increased from 6.6% (before 2005) to 20.2% (2011–15), but dropped to 6.5% (2016–20). Additionally, 32 clusters with shared pre-treatment HIVDR mutations were identified. The majority of clusters had R_0_ ≥ 1.0, indicating ongoing transmissions. The largest was a K103N cluster involving 16 MSM sequences sampled between 2010 and 2017, with an estimated time to the most recent common ancestor (tMRCA) of 2005 [95% higher posterior density (HPD), 2000–08], indicating propagation over 12 years.

**Conclusions:**

Compared to HETs, children and key populations had higher levels of pre-treatment HIVDR. Introduction of INSTIs after 2017 may have abrogated the increase in pre-treatment RTI mutations, albeit in the HET population only. Taken together, our findings underscore the need for targeted efforts towards equitable access to ART for children and key populations in Kenya.

## Introduction

By the end of 2020, an estimated 30 million individuals were receiving ART globally.^1^ The scale-up of ART has substantially reduced the rates of new HIV-1 infections, including vertical transmissions, HIV-1 related mortality, and improved life expectancy for millions of people with HIV (PWHIV).^1–3^ However, widespread use of ART has been associated with the emergence of HIV-1 drug resistance (HIVDR) and the transmission of drug-resistant viruses that can compromise therapy outcomes.^4^

The WHO recommends routine nationally representative HIVDR surveys to inform treatment strategies.^5^ Increasing levels of pre-treatment HIVDR to NNRTIs has been recognized as a problem for many years, especially in low- and middle-income countries (LMICs) where virological monitoring of patients on ART is suboptimal.^6,7^ In part, the increasing NNRTI resistance and poor tolerability to PIs informed WHO’s recommendation for a switch to integrase strand transfer inhibitors (INSTIs) as part of the first-line regimens in LMICs. INSTIs are more efficacious and have a high genetic barrier to resistance. Dolutegravir, a second-generation INSTI, is now widely adopted for both treatment-naive and experienced PWHIV.^8^ However, the potential impact of the switch to dolutegravir-based regimens, which came into effect in LMICs in 2017, on emergence and circulation of NNRTI-resistant strains is unclear.

Further, key populations including MSM, people who inject drugs (PWID), female sex workers (FSWs) and transgender people are disproportionately impacted by HIV-1 in sub-Saharan Africa (sSA). HIV-1 transmission from these key populations is thought to contribute disproportionately to Africa’s generalized HIV-1 epidemic.^9^ We previously demonstrated that the majority of HIV-1 transmissions in Kenya occur within distinct risk groups, and that transmission from heterosexuals (HETs) to key populations is more common than vice versa.^10,11^ However, it remains unknown whether there is differential transmission of HIVDR mutations among different risk groups. We aimed to describe prevalence, temporal trends and transmission linkages of pre-treatment HIVDR mutations within and between risk groups in Kenya.

## Methods

### Study population

HIV-1 partial *pol* sequences from Kenya were either newly generated from archived plasma samples (henceforth referred as newly generated) or retrieved from the Los Alamos HIV-1 sequence database (henceforth referred as published sequences).^12^ Newly generated HIV-1 *pol* sequences [approximately 1020 nucleotides (nt), HXB2 (K03455) positions 2267–3287] were generated from plasma obtained from the MSM Health Research Consortium. This is a multi-site collaboration between the Kenya Medical Research Institute-Wellcome Trust Research Programme (KWTRP) in Coastal Kenya,^13–15^ Nyanza Reproductive Health Society (NRHS) in Western Kenya,^16^ Sex Workers Outreach Program (SWOP) clinics in Nairobi, Kenya, the Targeted Research Advancing Sexual Health for Men who have Sex with Men (TRANSFORM) cohort from Nairobi, Kenya,^17^ and the national HIV-1 reference laboratory at the Kenya Medical Research Institute/Centre for Global Research (KEMRI/CGHR) in Kisumu, Kenya. Plasma samples from the collaboration were used to generate HIV-1 partial *pol* sequences as previously described.^18^ Published HIV-1 *pol* sequences of Kenyan origin were also retrieved from the Los Alamos HIV database on 20 March 2022.^12^

Sociodemographic, clinical and ART status data for newly generated sequences were obtained from the respective cohorts, while those from published sequences were extracted from referenced publications and their accompanying supplementary material. Where data for published sequences were missing, information was obtained through direct communication with study authors. Newly generated and published sequences were then annotated with sampling date, sampling location, risk group [HETs, MSM, PWID, FSWs and children (defined as individuals <18 years old)] and ART status (whether treatment naive or experienced). Where such information was still missing, related sequences were excluded from the analysis. Only sequences generated from ART-naive individuals were included in the analyses.

### HIV-1 subtype determination

Newly generated and published HIV-1 partial *pol* sequences were aligned with the HIV-1 Group M (subtypes A–K + recombinants) subtype reference sequences (http://www.hiv.lanl.gov) using the MAFFT algorithm in Geneious Prime 2019.^12^ Subtypes were determined based on a maximum-likelihood (ML) phylogenetic tree generated in PhyML using the general time-reversible substitution model with a gamma-distributed rate variation and proportion of invariant sites (GTR + Γ4 + Ι).^19^ Branch support was determined using the approximate likelihood ratio test with the Shimodaira–Hasegawa-like procedure (aLRT-SH) in PhyML and aLRT-SH ≥ 0.90 was considered significant. The phylogenetic tree was visualized using FigTree v1.4.4 (https://github.com/rambaut/figtree/releases), and subtypes assigned based on how sequences in our dataset clustered relative to the HIV-1 Group M reference sequences. Subtype assignment congruence was confirmed with the web-based subtyping algorithm COMET (available at https://comet.lih.lu) and REGA (version 3.46, https://www.genomedetective.com/app/typingtool/hiv). Unique recombinant forms (URFs) were resolved by boot-scan analysis in SimPlot.^20–22^

### Characterization of pre-treatment HIVDR

All sequences from antiretroviral-naive individuals were submitted to the Stanford HIV database (https://hivdb.stanford.edu) for determination and interpretation of HIVDR mutations. The Calibrated Population Resistance tool for protease and reverse transcriptase (PRRT) and for integrase (IN) sequences was used to identify pre-treatment HIVDR mutations based on the WHO list of mutations for the surveillance of transmitted HIVDR.^23,24^ Mutations were grouped and presented based on drug class as follows: NRTI, NNRTI, PI and INSTI mutations.

### Phylogenetic determination of clusters

HIV-1 sequences were grouped into subtype-specific datasets. For each index sequence, the 20 most similar sequences were obtained from the NCBI GenBank using the BLAST tool, as previously described.^25^ After removal of duplicate sequences from the same individual, the Kenyan sequences from our dataset and the reference sequences obtained from the BLAST search were compiled into subtype-specific alignments, excluding codon positions associated with drug-resistance mutations. ML phylogenies were generated in PhyML using the GTR + Γ4 + Ι model.^19^ For each subtype-specific phylogenetic tree, monophyletic clades with aLRT-SH support ≥ 0.9 and which were dominated (≥80%) by Kenyan sequences (compared with reference sequences) were defined as Kenyan HIV-1 clusters. Clusters were classified based on the number of sequences into dyads (two sequences), networks (3–14) and large clusters (>14 sequences).^25^ ML phylogenetic trees were annotated based on the most common mutations per drug class, i.e. K103NS, Y181CIV and G190AES (for NNRTI resistance mutations) and M184VI and T215 revs (for NRTI resistance mutations).

### Hierarchical phylogenetic modelling and Bayesian inference

Hierarchical phylogenetic models (HPMs) are useful for phylogenetic inferences when analysing a sparse sample.^26^ HPMs comprise two components: (i) an across-partition-level model, which captures the shared evolutionary history among all species in an analysis, often at a higher taxonomic level; and (ii) an individual partition model that provides more detailed information about evolutionary relationships within specific partitions. HPMs innovatively allow for the simultaneous fitting of both the across-partition-level model and individual partition models. Thus, information inferred at the broader taxonomic level can be shared as a prior with the individual partition models. This ‘feedback’ or sharing of information results in a borrowing of strength from one partition to another, which can lead to more accurate and precise estimates of evolutionary relationships.^26^

HPMs were incorporated in our phylodynamic analyses to date clusters. BEAST software (version 1.10.4) was used. The HKY nucleotide substitution model (unlinked for all partitions) was applied while selecting empirical base frequencies and a gamma site heterogeneity model with four gamma categories. Codon partitions were turned off. A strict clock model (unlinked for all partitions) was specified, and a constant size tree prior (with unlinked tree priors for all trees) maintained as a simple coalescent tree prior. We specified hierarchical priors for the substitution model parameters (on the log-normal scale for both ‘kappa’ and ‘alpha’ parameters, while setting the normal population mean hyperprior standard deviation to 5.0, as well as a gamma population precision hyperprior of initial value 1.0, shape value 0.01 and scale value 100.0). A second HPM was set for the clock rates (on the log-normal scale, with a normal population mean hyperprior standard deviation set to 100.0, and a gamma population precision hyperprior with an initial value 1.0, shape value 0.001 and scale value 1000.0). A final HPM was specified for the population sizes, setting the normal hyperprior standard deviation to 100.0, and the gamma hyperprior shape and scale to 0.001 and 1000.0, respectively. Markov chain Monte Carlo (MCMC) runs with a chain frequency of 300 million generations were computed in BEAST, logging every 300 000th iteration, and discarding the first 10% as chain burn-in. Convergence was determined in Tracer v.1.7.0 [defined as effective sample size (ESS) ≥ 100].^27^ Maximum clade credibility (MCC) trees were summarized from the posterior tree distribution using TreeAnnotator v1.10.4 (BEAST suite) and visualized using Figtree (v1.4.4, https://github.com/rambaut/figtree/releases).

Additional phylodynamic analyses were performed to estimate the population growth rate (r, years^−1^) per cluster using the GTR + Γ4 + Ι nucleotide substitution model, and a logistic growth coalescent tree prior.^28–30^ The basic reproductive number (R_0_, defined as the number of secondary infections that arise from a typical primary case in a completely susceptible population) per cluster was estimated based on the respective cluster growth rate I using the formula R_0_ = *r*D + 1 (where D represents the average duration of infectiousness, i.e. for individuals with uncontrolled viraemia who are more likely to transmit the virus, assuming uniform transmission rates over time).^28–30^ In the absence of empirical data on the duration of infectiousness in Kenya, R_0_ was modelled assuming varying D values (range, 1–8 years).

### Statistical analysis

Prevalence of pre-treatment HIVDR was determined as a proportion of sequences with at least one HIVDR mutation relative to the total number of sequences included in the analysis. Prevalence estimates were aggregated by calendar year of sampling into 5 year intervals to reflect major changes in treatment guidelines in Kenya as follows: before 2005 (before introduction of combination ART), 2005–10 (introduction of combination ART), 2011–15 (scale up of combination ART) and 2016–20 (introduction of INSTI-based regimens). Categorical data were compared with the χ² test and continuous data were compared with the Kruskal–Wallis test, where appropriate. Statistical differences between risk group and calendar year intervals were assessed using the Dunn’s test for multiple comparisons, with Bonferroni correction. Overall, drug class-specific, and mutation-specific HIVDR prevalence estimates and 95% CIs were presented. HIVDR temporal trends were assessed using *nptrends*, a non-parametric extension of the Wilcoxon rank-sum test.^31^ Data analysis and summary plots were done using Stata 15 (StataCorp LLC, College Station, TX, USA) and RStudio (version 1.2.5001) with the *ggplot2* package.^32^

### Nucleotide sequence accession numbers

Newly generated nucleotide sequences are available from GenBank under the accession numbers (MT084914–MT085076) and (OM109695–OM110282).

### Ethics

For newly generated sequences, informed consent for use of data and samples for research studies was obtained from participants under respective study protocols. Since published sequences were obtained from an open access repository, informed consent was not retrospectively obtained. Instead, science and ethics approval were obtained from the Kenya Medical Research Institute (KEMRI) Scientific and Ethics Review Unit (SERU 3547). All newly generated and published sequences were de-identified at source before inclusion in the analysis.

## Results

### Study population

**Figure 1.**
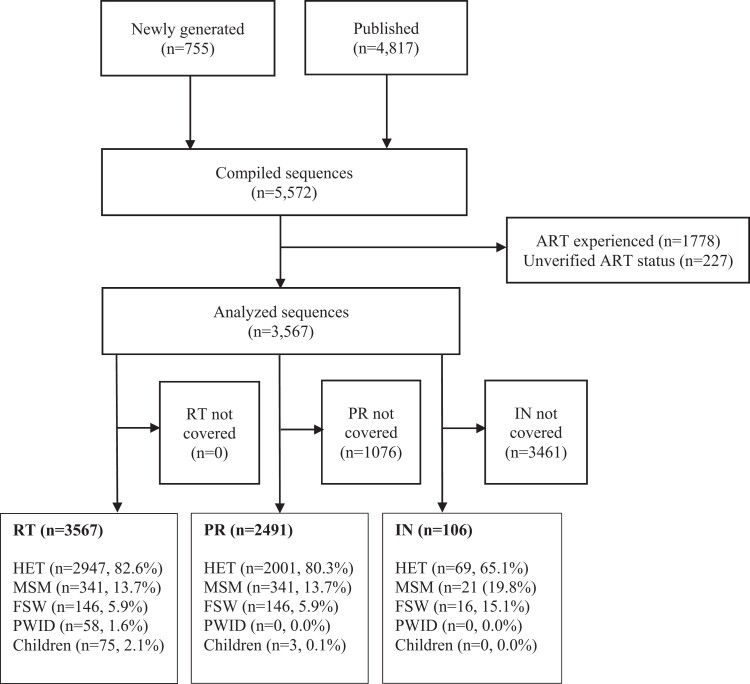
A flowchart summarizing the number of HIV-1 pol sequences (1986–2020) analysed in this study. RTI, reverse transcriptase inhibitor. The HET population was defined as at-risk men and women who did not report sex work or male same-sex behaviour.

**Figure 2.**
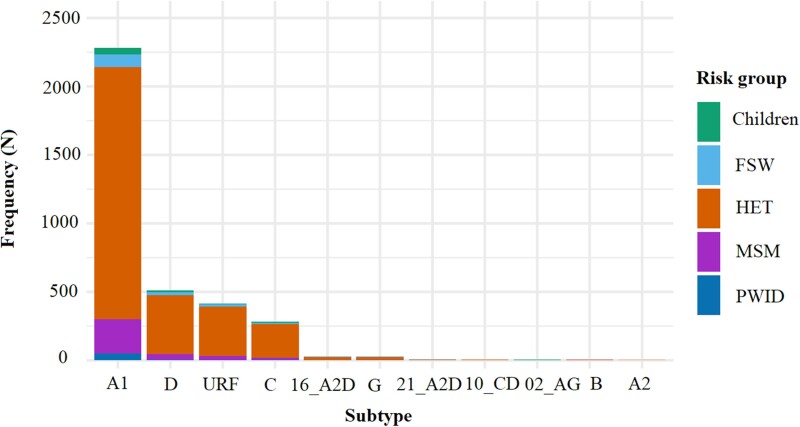
HIV-1 subtypes distribution within and between risk groups in Kenya (*n* = 3567; 1986–2020). A stacked bar graph summarizing subtype distribution in the full Kenyan HIV-1 sequence dataset. Subtypes are depicted on the *x*-axis and the frequency per risk group on the *y*-axis, coloured per risk group (green: children; sky blue: FSWs; vermillion: HETs; purple: MSM and dark blue: PWID). The most dominant subtypes were A1, D and C (and their recombinant forms) (*n* = 3567; 1986–2020).

Overall, 5572 HIV-1 *pol* sequences [newly generated (*n* = 755; Table S1, available as Supplementary data at *JAC* Online) and published (*n* = 4817)] sampled between 1986 and 2020 were considered. Of these, 3567 sequences were from ART-naive individuals and were included in the analysis (Figure 1). These comprised sequences covering the reverse transcriptase (*n* = 3567; 100.0%), protease (*n* = 2491; 69.8%) and IN (*n* = 106; 3.0%) coding regions. Most sequences were from the HET population (*n* = 2947; 82.6%), collected between 2006 and 2010 (*n* = 1997; 56.0%) and from the former Nairobi province (*n* = 1479; 41.5%) (Table 1). The most common circulating strain was HIV-1 sub-subtype A1 (*n* = 2282; 64.0%) (Figure 2), with subtype distribution being mostly similar across risk groups (Table S2).

**Table 1.** Distribution of newly generated and published HIV-1 *pol* sequences from antiretroviral therapy-naive individuals in Kenya (*n* = 3567; 1986–2020)

Characteristics		Newly generated *N* = 396 *n* (%)	Published *N* = 3171 *n* (%)	Total *N* = 3567 *n* (%)
Risk group	HETs	78 (19.7)	2869 (90.5)	2947 (82.6)
	MSM	190 (48.0)	151 (4.8)	341 (9.6)
	PWID	0 (0.0)	58 (1.8)	58 (1.6)
	FSWs	128 (32.3)	18 (0.6)	146 (4.1)
	Children	0 (0.0)	75 (2.4)	75 (2.1)
Year (range)	Before 2005	18 (4.5)	433 (13.7)	451 (12.6)
	2006–10	110 (27.8)	1887 (59.5)	1997 (56.0)
	2011–15	41 (10.4)	842 (26.6)	883 (24.8)
	2016–20	227 (57.3)	9 (0.3)	236 (6.6)
Province	Nairobi	150 (37.9)	1329 (41.9)	1479 (41.5)
	Coast	175 (44.2)	852 (26.9)	1027 (28.8)
	Nyanza	71 (17.9)	662 (20.9)	733 (20.5)
	Rift Valley	0 (0.0)	294 (9.3)	294 (8.2)
	Central	0 (0.0)	33 (1.0)	33 (0.9)
	North-Eastern	0 (0.0)	1 (0.0)	1 (0.0)

HET is defined as presumed heterosexual, i.e. at-risk men and women not reporting sex work or male same-sex behaviour.

### Prevalence of pre-treatment HIVDR

**Figure 3.**
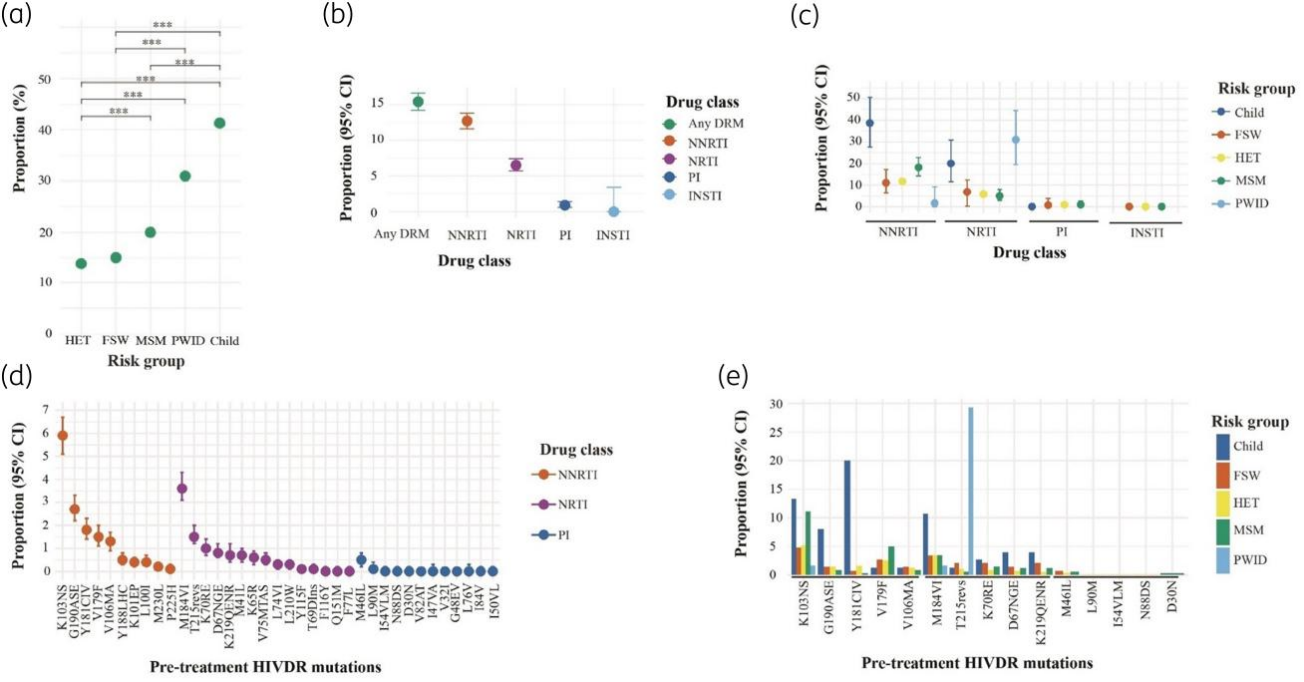
Distribution of pre-treatment HIVDR by: (a) proportion of individuals with at least one pre-treatment HIVDR mutation by risk groups, with pairwise comparisons and Bonferroni corrections; (b) proportion of individuals with at least one pre-treatment HIVDR mutation by drug class (with 95% CI); (c) proportion of individuals with at least one pre-treatment HIVDR mutation by both risk group and drug class (with 95% CI); (d) proportion of pre-treatment HIVDR mutations by drug class (with 95% CI); and (e) proportion of the five most prevalent pre-treatment HIVDR mutations per drug class distributed by risk groups, in Kenya (*n* = 3567; 1986–2020).

Of 3567 sequences, 550 (15.4%, 95% CI: 14.2–16.6) had at least one pre-treatment HIVDR mutation. Overall, pre-treatment HIVDR mutations were most common amongst children (*n* = 31; 41.3%), followed by PWID (*n* = 18; 31.0%), MSM (*n* = 68; 19.9%), FSWs (*n* = 22; 15.1%) and HETs (*n* = 411; 13.9%), with the distribution differing significantly across risk groups (Figure 3a, Table S3 and Table S4). NNRTI resistance mutations were most common [*n* = 453; 12.7% (95% CI: 11.6–13.8)], followed by NRTI [*n* = 232; 6.5% (95% CI: 5.7–7.4)] and PIs [*n* = 23; 0.9% (95% CI: 0.6–1.4)]. There were no INSTI resistance mutations observed (Figure 3b, Table S3 and Table S4). NNRTI resistance mutations were highest among children (*n* = 29; 38.7%), followed by MSM (*n* = 62; 18.2%), HETs (*n* = 345, 11.7%) and FSWs (*n* = 16; 11.0%). NRTI resistance mutations were highest among PWID (*n* = 18; 31.0%), followed by children (*n* = 15; 20.0%), FSWs (*n* = 10; 6.8%), HETs (*n* = 172; 5.8%) and MSM (*n* = 17; 5.0%). PI resistance mutations were <1.0% in HETs, FSWs and MSM, and none were observed in children and PWID (Figure 3c).

Of sequences with any pre-treatment HIVDR, the most common pre-treatment NNRTI, NRTI and PI drug-resistance mutations were K103NS (*n* = 210; 5.9%), M184VI (*n* = 130; 3.6%) and M46IL (*n* = 12; 0.5%), respectively (Figure 3d and Table S5). The most common mutations by risk group were the T215revs (for PWID), Y181CIV (for children) and K103NS (for HETs, MSM and FSWs) (Figure 3e).

### Temporal trends in pre-treatment HIVDR

**Figure 4.**
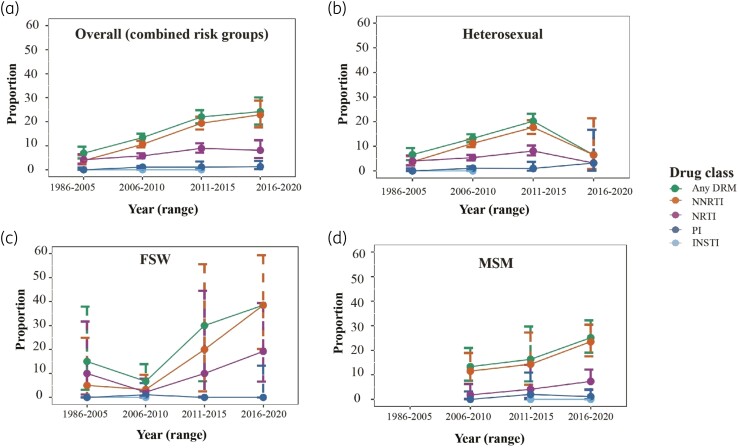
Temporal trends in pre-treatment HIVDR in Kenya among (a) overall ART-naive individuals (all risk groups combined); (b) HETs in the general population; (c) FSWs; and (d) MSM. Proportions are presented with error bars representing 95% CIs as estimated by a non-parametric linear-by-linear test for trends *(nptrends*) (*n* = 3567; 1986–2020).

Overall, there was an increase in pre-treatment HIVDR from 6.9% (before 2005) to 13.4% (2011–15), 22.0% (2011–15) and 24.2% (2016–20) (*P* < 0.001) (Figure 4a, Table S6 and Table S7). NNRTI resistance increased from 3.8% (before 2005) to 22.9% (2016–20, *P*< 0.001) while NRTI resistance increased from 4.2% (before 2005) to 8.1% (2016–20, *P* = 0.061). PI resistance remained stable below 2% throughout the calendar periods (*P* = 0.298).

Amongst the HET population, pre-treatment HIVDR increased from 6.6% (before 2005) to 13.2% (2006–10), 20.2% (2011–15), followed by a significant decline to 6.5% (2016–20) (*P* < 0.001) (Figure 4b, Table S6 and Table S7). Levels of NNRTI and NRTI resistance also increased from 3.7% and 4.0% (before 2005) to 17.7% and 8.1% (2011–15), followed by a decline to 6.5% and 3.2% (2015–20, *P* < 0.001 and *P* = 0.079, respectively). PI resistance remained low at 0.0% (before 2005), 1.0% (2011–15) and 3.2% (2016–20, *P* = 0.839).

Among FSWs, NNRTI resistance also increased significantly between 2006–10 and 2015–20 (*P* = 0.001) (Figure 4c, Table S6 and Table S7). Among MSM there was an overall increase in NNRTI and NRTI resistance between 2006–10 and 2015–20, though this did not achieve statistical significance (*P* = 0.399 and *P* = 0.241, respectively) (Figure 4d, Table S6 and Table S7). Data on PWID and children fell within one calendar year period (2006–10 and 2011–15, respectively), and were therefore excluded from trend analysis.

### Phylogenetic clustering of sequences with shared pre-treatment HIVDR mutations

Overall, 491 sequences with pre-treatment HIVDR mutations were analysed, comprising HIV-1 sub-subtype A1 (*n* = 377), subtype C (*n* = 31) and subtype D (*n* = 83). Phylogenetic analysis revealed 32 clusters (size range, 2–16 sequences) with shared mutations K103NS (*n* = 10; 31.3%), Y181CIV (*n* = 6; 18.8%), M184VI (*n* = 5; 15.6%), G190AES (*n* = 3; 9.4%), T215revs (*n* = 4; 12.5%), K103NS/M184VI (*n* = 3; 9.4%) and K103NS/K101P (*n* = 1; 3.1%). Clusters included dyads (*n* = 26; 81.3%), networks (*n* = 4; 12.5%) and large clusters (*n* = 1; 3.1%). Clusters were either risk-group exclusive, i.e. HETs only (*n* = 24; 75.0%), MSM only (*n* = 2; 6.3%) and PWID only (*n* = 1; 3.1%) or mixed clusters including HETs/MSM (*n* = 2; 6.3%), HETs/FSWs (*n* = 1; 3.1%) and HETs/children (*n* = 2; 6.3%) (Table 2).

**Table 2.** Characteristics of clusters (n = 32) with shared pre-treatment HIVDR mutations and distributed into subtypes and risk group

HIVDR mutation	Cluster number	subtype	Number of tips (*n*)	Risk group
G190AES	#1	A1	2	HETs/children
	#2	A1	2	HETs
	#3	A1	2	HET/FSWs
K103NS	#4	A1	2	HETs
	#5	A1	2	HETs/MSM
	#6	A1	2	HETs/MSM
	#7	A1	3	MSM
	#8	A1	16	MSM
	#9	D	2	HETs
	#10	D	2	HETs
	#11	D	2	HETs
	#12	D	2	HETs
	#13	D	2	HETs
M184VI	#14	A1	2	HETs
	#15	A1	2	HETs
	#16	A1	2	HETs
	#17	D	2	HETs
	#18	D	2	HETs
T215revs	#19	A1	2	HETs
	#20	A1	2	HETs
	#21	A1	11	PWID
	#22	D	2	HETs/children
Y181CIV	#23	A1	2	HETs
	#24	A1	2	HETs
	#25	A1	2	HETs
	#26	D	6	HETs
	#27	D	2	HETs
	#28	D	2	HETs
K103NS/M184VI	#29	A1	2	HETs
	#30	A1	2	HETs
	#31	A1	2	HETs
K103N/K101P	#32	A1	3	HETs

Number of clusters with the most dominant pre-treatment HIVDR mutations per drug class i.e. NNRTI (K103NS, Y181CIV, G190AES) and NRTI (M184VI and T215revs). Two clusters shared more than one mutation (K103NS/M184VI and K103N/K101P). HET is defined as presumed heterosexual, i.e. at-risk men and women not reporting sex work or male same-sex behaviour.

**Figure 5.**
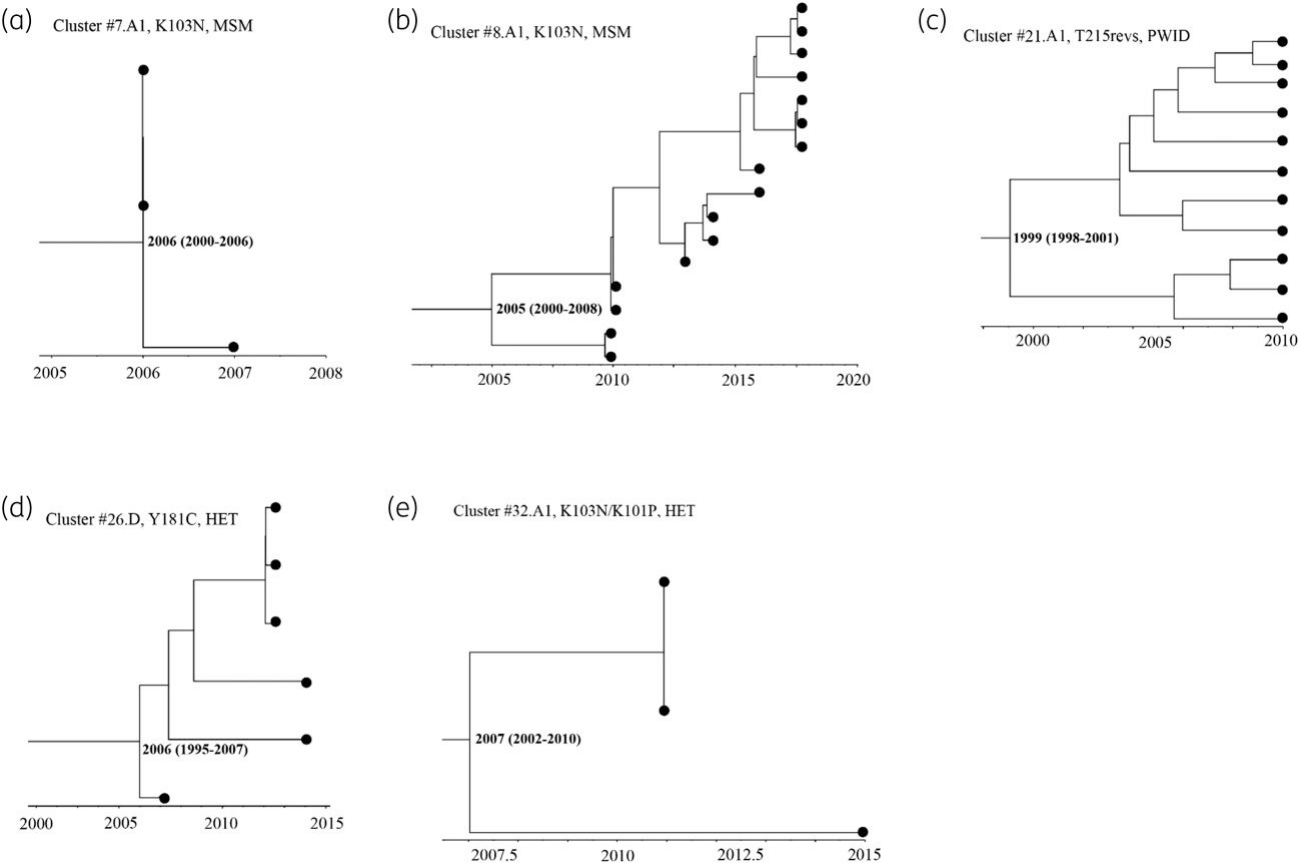
MCC trees estimated using a Bayesian MCMC approach showing Kenyan HIV clusters (≥3 sequences) with shared pre-treatment HIVDR mutations. For each cluster, the estimated time of origin (95% HPD intervals) is indicated at the root node, and branch tips correspond to sampling dates per sequence. Legends correspond to cluster number (#), subtype (A1 or D), mutation and risk group.

Bayesian dating was performed for networks and large clusters, which included four sub-subtype A1 and one subtype D clusters (Table 3 and Figure 5). The largest comprised 16 MSM with the K103N mutation. This cluster had an estimated time to the most recent common ancestor (tMRCA) of 2005 [95% higher posterior density (HPD), 2000–08], where the most recent sample was collected in 2017, suggesting that this lineage had persisted over 12 years [growth rate = 0.10/year and R_0_ = 1.10 (95% HPD, −5.08–11.28)]. Another long-standing cluster involved 11 PWID having the T215rev mutation with an estimated tMRCA of 1999 (95% HPD, 1998–01), where the most recent sample was collected in 2010, indicating that this lineage may have persisted for over 11 years [growth rate = 0.38/year, R_0_ = 1.38 (95% HPD, 1.00–3.91)]. Overall, the majority of clusters had basic reproductive number, R_0_ ≥ 1.0 (assuming D values ranging from 1 to 8 years), which may be indicative of ongoing propagation of pre-treatment HIVDR mutations among untreated individuals in Kenya (Table 3 and Table S8).

**Table 3.** Characteristics of clusters having ≥3 sequences (*n* = 5) with shared pre-treatment HIVDR mutations

Cluster	HIVDR mutation	Subtype	Tips (*N*)	*tMRCA (95% HPD)	Sampling period	Growth rate (95% HPD)	R_0_ (where D = 1) (95% HPD)	Risk group
#7.A1	K103N	A1	3	2006 (2000–2006)	2006–07	−0.06 (−4.21–0.70)	0.94 (−3.21 to 1.70)	MSM
#8.A1	K103N	A1	16	2005 (2000–2008)	2010–17	0.10 (−6.08–10.28)	1.10 (−5.08 to 11.28)	MSM
#21.A1	T215revs	A1	11	1999 (1998–2001)	2010	0.38 (0.00–2.91)	1.38 (1.00–3.91)	PWID
#26.D	Y181C	D	6	2006 (1995–2007)	2007–13	0.00 (0.00–4.37)	1.00 (1.00–5.37)	HETs
#32.A1	K103N/K101P	A1	3	2007 (2002–2010)	2011–15	0.94 (0.00–6.72)	1.94 (1.00–7.72)	HETs

Bayesian dating was restricted to five clusters having ≥3 sequences and with the three most dominant pre-treatment HIVDR mutations per drug class, i.e. NNRTI (K103N and Y181C) and NRTI mutations (T215revs). One cluster shared more than one mutation (K103N/K101P). HET is defined as at-risk men and women who did not report sex work or male same-sex behaviour.

## Discussion

We used HIV-1 *pol* sequence data spanning a period of more than three decades to assess the prevalence, temporal trends and transmission of pre-treatment HIVDR among different risk groups in Kenya. Overall prevalence of pre-treatment HIVDR was high (15.4%), which was largely a reflection of the high levels of pre-treatment NNRTI drug-resistance mutations. Notably, there was comparatively lower (<10%) pre-treatment NRTI drug resistance and no pre-treatment INSTI resistance in the study population, albeit based on a limited number of HIV-1 IN gene sequences mostly collected prior to the transition to INSTI-based regimens in Kenya. Taken together, these observations justify the retention of two NRTIs and the switch to a dolutegravir-based regimen for HIV-1 treatment in Kenya.^33^

We observed an increasing trend in pre-treatment HIVDR at a countrywide level, which was also largely driven by NNRTI resistance. This is consistent with previous subnational data from Kenya and data from elsewhere in the global context.^6,7,34–36^ NNRTIs have a low genetic barrier to resistance, which permits outgrowth of resistant variants under suboptimal drug pressure, and mutations may persist for long durations, facilitating their onward transmission.^37,38^ Of interest, pre-treatment NNRTI and NRTI resistance among HETs (the largest risk group in this study, which is representative of the HIV-1 epidemic in Kenya) increased between 2005 and 2015 but declined between 2015 and 2020. This coincides with the nationwide transition from NNRTI to INSTI-based ART regimens,^33^ suggesting that scale-up of dolutegravir-based regimens may have abrogated emergence and circulation of pre-treatment NNRTI and NRTI resistance mutations. In contrast, pre-treatment HIVDR among FSWs and MSM increased consistently through to 2015–20. Interestingly, as of 2020, ART coverage was lower among key populations: 73% in FSWs, 68% in PWID and 63% in MSM, compared with 86% in the general HET population.^39^ Taken together, these observations may explain the higher resistance levels observed in key populations compared with the HET population, and warrants further investigation.

Our observations are consistent with findings from a WHO-led systematic review reporting higher levels of pre-treatment HIVDR among key populations compared with the HET population in the global context (with better sampling densities from higher-income countries, but with limited representation of data from key populations in Africa).^5^ With the transition to INSTI-based regimens, enhanced monitoring of INSTI resistance using contemporary sequences from children, HETs and key populations would be prudent to inform future treatment strategies in Kenya.

We observed high levels of K103NS, Y181CIV and G190AES mutations, which likely reflect extensive selection in persons receiving nevirapine and efavirenz, both of which comprised the main NNRTI options for first-line regimens historically in Kenya.^40^ This observation was more evident among children, who had the highest prevalence of pre-treatment HIVDR, with NNRTI-associated mutations Y181CIV and K103NS being the most common, which is consistent with literature.^41,42^ These mutations likely point towards the widespread use of nevirapine for prevention of mother-to-child transmission (pMTCT). Due to a paucity of HIV-1 sequence data from children during the 2016–20 time interval, temporal trend analyses were not possible. Continued surveillance of pre-treatment HIVDR in children to assess the impact, if any, of the transition to a dolutegravir-based regimen on NNRTI-associated mutations in Kenya is therefore warranted.

We observed several HIV-1 clusters with shared mutations spanning more than 10 years, indicating onward propagation of HIVDR among treatment-naive individuals as new infections, which has also been reported from developed settings.^43^ There was more frequent HIV-1 clustering of K103NS strains, indicating extensive transmission of this pre-treatment mutation in Kenya, possibly dating back to the single-dose nevirapine era. Most of the clusters had a basic reproductive number, R_0_ ≥ 1.0, suggesting ongoing propagation of NRTI and NNRTI HIVDR transmissions. Assessment of these trends over the next years is needed to determine whether roll-out of the more efficacious INSTIs as first-line regimens will terminate circulation of NNRTI resistance mutations in Kenya.

The main strength of our study was the analysis of sequence data from multiple geographically diverse regions from Kenya and spanning over three decades. However, the study is not without limitations, most of which are consistent with population-level studies that leverage secondary sources of data. First, although we included all the Kenyan HIV-1 *pol* sequences available in the public domain and complemented the sample size by generating more sequences from well-characterized cohorts, the distribution of sequences was still sparse in some risk groups during some time intervals. This may have resulted in potential sampling bias, which may have impacted our temporal trends and phylodynamic inferences, and warrants cautious interpretation of our findings. Second, we extracted a limited set of demographic and clinical data including year of sampling, risk group and ART status. This implies that we were not able to control for other factors like duration from the estimated date of infection and unreported history of pre-exposure prophylaxis use. While it would have been ideal to control for these factors in our analyses, most are usually either not available or not systematically collected across parent studies.

In conclusion, we demonstrated an increase in pre-treatment HIVDR over the last two decades, which justifies the switch to INSTI-based therapy. Importantly, we show that children and key populations have significantly higher levels of pre-treatment HVIDR compared with the general HET population in Kenya. Remarkably, INSTI-based regimens may have abrogated propagation of RTI mutations among the general HET population, but not in hard-to-reach key populations. Our study further underscores the challenges with access to HIV care and treatment for key populations, as demonstrated with increasing pre-treatment HIVDR in MSM and FSWs, despite the broad nationwide introduction of INSTI-based regimens. Using phylodynamic analyses, we also demonstrate long-standing and ongoing propagation of pre-treatment HIVDR strains, that were mostly risk-group exclusive. Taken together, our findings underscore need for targeted efforts towards equitable access to ART for children and key populations in Kenya.

## Supplementary Material

dkad375_Supplementary_Data

## Supplementary data

Tables S1 to S8 are available as Supplementary data at *JAC* Online.
