# Supplementary material for: Temporal trends and transmission dynamics of pre-treatment HIV-1 drug resistance within and between risk groups in Kenya, 1986–2020
Source: J Antimicrob Chemother. 2023 Dec 13;79(2):287–96. doi: 10.1093/jac/dkad375 (PMC10832587; doi:10.1093/jac/dkad375)
Supplement: dkad375_Supplementary_Data [file dkad375_supplementary_data.pdf]

SUPPLEMENTARY MATERIAL

TEMPORAL TRENDS AND TRANSMISSION DYNAMICS OF PRE-TREATMENT HIV-1 DRUG RESISTANCE WITHIN AND BETWEEN RISK GROUPS IN KENYA, 1986-2020.

George M. NDUVA[1,2*], Frederick OTIENO[3], Joshua KIMANI[4,5], Yiakon SEIN[2], Dawit A. ARIMIDE[1], Lyle R. MCKINNON[4,5,6], Francois CHOLETTE[5,7], Morris K. LAWRENCE[8], Maxwell MAJIWA[9], Moses MASIKA[10], Gaudensia MUTUA[10], Omu ANZALA[10], Susan M. GRAHAM[2,11], Larry GELMON[4,5], Matt A. PRICE[12,13], Adrian D. SMITH[14], Robert C. BAILEY[3,15], Patrik MEDSTRAND[1], Eduard J. SANDERS[2,14#], Joakim ESBJÖRNSSON[1,14#], and Amin S. HASSAN[1,2#*].

[#]Equal contribution as senior authors

[1]Lund University, Lund, Sweden; [2]KEMRI/Wellcome Trust Research Programme, Kilifi, Kenya; [3]Nyanza Reproductive Health Society, Kisumu, Kenya; [4]University of Nairobi, Nairobi, Kenya; [5]University of Manitoba, Winnipeg, Canada; [6]Centre for the AIDS Programme of Research in South Africa (CAPRISA), South Africa; [7]National Microbiology Laboratory at the JC Wilt Infectious Diseases Research Centre, Public Health Agency of Canada, Winnipeg, Canada; [8]Pwani University, Kilifi, Kenya; [9]KEMRI/Centre for Global Health Research, Kisumu, Kenya; [10]KAVI Institute of Clinical Research, University of Nairobi, Nairobi, Kenya; [11]University of Washington, Seattle, USA; [12]IAVI, New York, USA; [13]University of California, San Francisco, USA; [14]University of Oxford, Oxford, United Kingdom; and [15]University of Illinois at Chicago, USA.

Files in this Data Supplement:

Table S1. A summary scheme of newly generated sequences by cohort in the study.

Table S2. HIV-1 subtypes distribution within-and-between risk groups in Kenya (n=3567, 1986-2020).

Table S3. Frequencies of pre-treatment HIV drug resistance by drug classes and risk groups in Kenya (proportions and 95% confidence intervals [95% CI]) (n=3567, 1986-2020).

Table S4. A summary of Dunn's post hoc test of multiple comparisons using rank sums indicating the differences in overall, nucleoside-, and non-nucleoside reverse transcriptase inhibitor mutations between HIV-1 risk groups in Kenya (n=3567, 1986-2020).

Table S5. Frequency and proportion of drug class-specific pre-treatment HIVDR mutations among sequences from treatment naïve with any pre-treatment HIVDR mutation by drug class and risk group among ART-naïve individuals in Kenya (n=3567, 1986-2020).

Table S6. Temporal trends in HIV-1 drug resistance among ART naïve individuals with different risk groups, including the frequencies, (proportions and 95% confidence intervals [95% CI]) in Kenya (n=3567, 1986-2020).

Table S7. A summary of Dunn's post hoc test of multiple comparisons using rank sums indicating the temporal differences in nucleoside and non-nucleoside reverse transcriptase inhibitor mutations among different HIV-1 risk groups in Kenya.

Table S8. Estimated R_0 values of clusters having ≥3 sequences (n=5) with shared pre-treatment HIVDR mutations.

Table S1. A summary of all newly generated sequences by cohort site in the study (N=755).

**Site**	**Risk group**			
	**HET**	**MSM**	**FSW**	**PWID**
**KWTRP**	48	21	107	0
**NHRS**	0	57	14	0
**KAVI-ICR**	30	0	7	0
**SWOP**	0	50	0	0
**TRANSFORM**	0	85	0	0
**KEMRI-CGHR**	336	0	0	0
**Total**	414	213	128	0

Abbreviations: HET, heterosexual adults; MSM, men who have sex with men; FSW, female sex workers; PWID, people who inject drugs. Site abbreviations: KWTRP, Kenya Medical Research Institute (KEMRI) -Wellcome Trust (Coastal Kenya); NHRS, Nyanza Reproductive Health Society (in Western Kenya); KAVI-ICR, Kenya AIDS Vaccine Initiative's Institute of Clinical Research (in Nairobi, Central Kenya); SWOP, Sex Workers Outreach Program clinics in Nairobi, TRANSFORM, a cohort of transfeminine people and cisgender men who have sex with men in Nairobi; KEMRI-CGHR, Kenya Medical Research Institute (KEMRI) – Centre for Global Health Research (Western Kenya).

Table S2. HIV-1 subtypes distribution within-and-between risk groups in Kenya (n=3567, 1986-2020).

**Subtype**	**HET** **n=2,947 (%)**	**MSM** **n=341 (%)**	**FSW** **n=146 (%)**	**PWID** **n=58 (%)**	**Children** **n=75 (%)**	**Total** **n=3,567 (%)**
A1	1840 (62.4%)	250 (73.3%)	94 (64.4%)	51 (87.9%)	47 (62.7%)	2282 (64.0%)
D	431 (14.6%)	42 (12.3%)	20 (13.7%)	3 (5.2%)	13 (17.3%)	509 (14.3%)
URF	362 (12.3%)	31 (9.1%)	14 (9.6%)	1 (1.7%)	5 (6.7%)	413 (11.6%)
C	245 (8.3%)	16 (4.7%)	13 (8.9%)	2 (3.5%)	6 (8.0%)	282 (7.9%)
16_A2D	25 (0.9%)	0 (0.0%)	4 (2.7%)	0 (0.0%)	1 (1.3%)	30 (0.8%)
G	24 (0.8%)	1 (0.3%)	1 (0.7%)	0 (0.0%)	1 (1.3%)	27 (0.8%)
21_A2D	6 (0.2%)	0 (0.0%)	0 (0%)	1 (1.7%)	1 (1.3%)	8 (0.2%)
10_CD	6 (0.2%)	0 (0.0%)	0 (0.0%)	0 (0.0%)	0 (0.0%)	6 (0.2%)
02_AG	3 (0.1%)	0 (0.0%)	0 (0.0%)	0 (0.0%)	1 (1.3%)	4 (0.1%)
B	3 (0.1%)	1 (0.3%)	0 (0.0%)	0 (0.0%)	0 (0.0%)	4 (0.1%)
A2	2 (0.1%)	0 (0.0%)	0 (0.0%)	0 (0.0%)	0 (0.0%)	2 (0.1%)

Abbreviations: MSM, men who have sex with men; PWID, people who inject drugs; FSW, female sex worker; HET, presumed heterosexual i.e., men and women not reporting sex work or male same-sex behaviour; URF, unique recombinant forms; CRF, circulating recombinant form; ART, anti-retroviral therapy.

Table S3. Frequencies of pre-treatment HIV drug resistance by drug classes and risk groups in Kenya (proportions and 95% confidence intervals [95% CI]) (n=3567, 1986-2020).

**Drug class**	**Overall (n=3567)**	**HET (n=2947)**	**MSM (n=341)**	**FSW (n=146)**	**PWID (n=58)**	**Children (n=75)**
Any HIVDR (n=3567)	550/3567 (15.4 [14.2-16.6])	411/2947 (13.9 [12.7-15.2])	68/341 (19.9 [15.8-24.6])	22/146 (15.1 [9.7-21.9])	18/58 (31.0 [19.5-44.5])	31/75 (41.3 [30.1-53.3])
NNRTI (n=3567)	453/3567 (12.7 [11.6-13.8])	345/2947 (11.7 [10.6-12.9])	62/341 (18.2 [14.2-22.7])	16/146 (11.0 [6.4-17.2])	1/58 (1.7 [0-9.2])	29/75 (38.7 [27.6-50.6])
NRTI (n=3567)	232/3567 (6.5 [5.7-7.4])	172/2947 (5.8 [5.0-6.7])	17/341 (5.0 [2.9-7.9])	10/146 (6.8 [0.3-12.3])	18/58 (31.0 [19.5-44.5])	15/75 (20.0 [11.6-30.8])
PI (n=2491)	23/2491 (0.9 [0.6-1.4])	19/2001 (0.9 [0.6-1.5])	3/341 (0.9 [0.1-2.5])	1/146 (0.7 [0-3.8])	(N/A)	0/3 (0.0 [0-70.8])
INSTI (n=106)	0/106 (0.0 [0.0-3.4*])	0/69 (0.0 [0.0-0.5*])	0/21 (0.0 [0.0-16.1*])	0/16 (0.0 [0.0-20.6*])	(N/A)	(N/A)

*Abbreviations: ART, antiretroviral therapy; HET, presumed heterosexual i.e., men and women not reporting sex work or male same-sex behaviour; MSM, men who have sex with men; PWID, people who inject drugs; FSW, female sex worker; NRTI, nucleoside reverse transcriptase inhibitors; NNRTI, non-nucleoside reverse transcriptase inhibitors; PI, protease inhibitors; INSTI, integrase strand transfer inhibitors. *One-sided, 97.5% confidence interval.*

Table S4. A summary of Dunn's post hoc test of multiple comparisons using rank sums indicating the differences in overall, nucleoside-, and non-nucleoside reverse transcriptase inhibitor mutations between HIV-1 risk groups in Kenya (n=3567, 1986-2020).

	**Any HIVDR mutation**		**NNRTI**		**NRTI**	
**Risk Group comparison**	**Mean rank difference**	**P-value (adjusted)**	**Mean rank difference**	**P-value (adjusted)**	**Mean rank difference**	**P-value (adjusted)**
MSM-HET	2.9	0.019	3.4	0.00	-0.6	1.000
FSW-HET	0.4	1.000	-0.3	1.00	0.5	1.000
PWID-HET	3.6	0.002	-2.3	0.12	7.7	0.000
Child-HET	6.5	0.000	6.9	0.00	4.9	0.000
FSW-MSM	-1.4	0.863	-2.2	0.14	0.8	1.000
PWID-MSM	2.2	0.153	-3.5	0.00	7.4	0.000
Child-MSM	4.6	0.000	4.8	0.00	4.8	0.000
PWID-FSW	2.8	0.022	-1.8	0.37	6.3	0.000
Child-FSW	5.1	0.000	5.9	0.00	3.8	0.001
Child-PWID	1.6	0.515	6.3	0.00	-2.6	0.053

Abbreviations: NNRTI, non-nucleoside reverse transcriptase inhibitor; NRTI, nucleoside reverse transcriptase inhibitor; MSM, men who have sex with men; PWID, people who inject drugs; FSW, female sex worker; HET, at-risk men and women who did not report sex work or male same-sex behaviour; Child, children infected perinatally.

Table S5. Frequency and proportion of drug class-specific pre-treatment HIVDR mutations among sequences from treatment naïve with any pre-treatment HIVDR mutation by drug class and risk group among ART-naïve individuals in Kenya (n=3567, 1986-2020).

**Drug class**	**HIVDR mutation**	**Number and proportion**
NNRTI	K103NS	210 (5.9%)
	G190ASE	98 (2.7%)
	Y181CIV	65 (1.8%)
	V179F	55 (1.5%)
	V106MA	45 (1.3%)
	Y188LHC	17 (0.5%)
	K101EP	16 (0.4%)
	L100I	13 (0.4%)
	M230L	8 (0.2%)
	P225H	5 (0.1%)
NRTI	M184VI	130 (3.6%)
	T215revs	56 (1.6%)
	K70RE	36 (1.0%)
	D67NGE	30 (0.8%)
	K219QENR	24 (0.7%)
	M41L	20 (0.6%)
	K65R	18 (0.5%)
	V75MTAS	10 (0.3%)
	L74VI	9 (0.3%)
	L210W	5 (0.1%)
	Y115F	3 (0.1%)
	T69Dins	3 (0.1%)
	F116Y	2 (0.1%)
	Q151M	2 (0.1%)
	F77L	1 (0.0%)
PI	M46IL	12 (0.5%)
	L90M	3 (0.1%)
	I54VL	2 (0.1%)
	N88DS	2 (0.1%)
	D30N	2 (0.1%)
	V82A	1 (0.0%)
	I47VA	1 (0.0%)
	V32I	1 (0.0%)
	G48E	1 (0.0%)
INSTI	None	None

Abbreviations: ART, anti-retroviral therapy; NRTI, nucleoside reverse transcriptase inhibitors; NNRTI, non-nucleoside reverse transcriptase inhibitors; PI, protease inhibitors; INSTI, integrase strand transfer inhibitors.

Table S6. Temporal trends in HIV-1 drug resistance among ART naïve individuals with different risk groups, including the frequencies, (proportions and 95% confidence intervals [95% CI]) in Kenya (n=3567, 1986-2020).

Risk group	Years	Any HIVDR (n=3567)	NNRTI (n=3567)	NRTI (n=3567)	PI (n=2491)	INSTI (n=106)
Overall	Before 2005 (n=451)	31/451 (6.9 [4.7-9.6])	17/451 (3.8 [2.2-6])	19/451 (4.2 [2.6-6.5])	0/391 (0.0 [0.0-0.9*])	0/60 (0.0 [0.0-6.0*])
	2006-2010 (n=1997)	268/1997 (13.4 [12-15])	211/1997 (10.6 [9.3-12])	115/1997 (5.8 [4.8-6.9])	17/1611 (1.1 [0.6-1.6])	0/42 (0.0 [0.0-8.4*])
	2011-2015 (n=883)	194/883 (22.0 [19.3-24.8])	171/883 (19.4 [16.8-22.1])	79/883 (8.9 [7.1-11])	3/253 (1.1 [0.2-3.4])	0/4 (0.0 [0.0-60.2*])
	2016-2020 (n=236)	57/236 (24.2 [18.8-30.1])	54/236 (22.9 [17.7-28.8])	19/236 (8.1 [4.9-12.3])	3/236 (1.3 [0.3-3.7])	(N/A)
HET	Before 2005 (n=427)	28/427 (6.6 [4.4-9.3])	16/427 (3.7 [2.2-6])	17/427 (4.0 [2.3-6.3])	0/368 (0.0 [0.0-1.0])	0/52 (0.0 [0.0-6.8*])
	2006-2010 (n=1736)	229/1736 (13.2 [11.6-14.9])	194/1736 (11.2 [9.7-12.8])	93/1736 (5.4 [4.3-6.5])	16/1408 (1.1 [0.7-1.8])	0/17 (0.0 [0.0-19.5*])
	2011-2015 (n=753)	152/753 (20.2 [17.4-23.2])	133/753 (17.7 [15-20.6])	61/753 (8.1 [6.3-10.3])	2/194 (1.0 [0.1-3.7])	(N/A)
	2016-2020 (n=31)	2/31 (6.5 [0.8-21.4])	2/31 (6.5 [0.8-21.4])	1/31 (3.2 [0.1-16.7])	1/31 (3.2 [0.1-16.7])	(N/A)
MSM	Before 2005 (n=0)	(N/A)	(N/A)	(N/A)	(N/A)	(N/A)
	2006-2010 (n=113)	15/113 (13.3 [7.6-20.9])	13/113 (11.5 [6.3-18.9])	2/113 (1.8 [0.2-6.2])	0/113 (0.0 [0.0-3.2*])	(N/A)
	2011-2015 (n=49)	8/49 (16.3 [7.3-29.7])	7/49 (14.3 [5.9-27.2])	2/49 (4.1 [0.5-14])	1/49 (2 [0.1-10.9])	0/13 (0.0 [0.0-24.7*])
	2016-2020 (n=179)	45/179 (25.1 [19-32.2])	42/179 (23.5 [17.5-30.4])	13/179 (7.3 [3.9-12.1])	2/179 (1.1 [0.1-4.0])	0/1 (0.0 [0.0-97.5*])

PWID	Before 2005 (n=0)	(N/A)	(N/A)	(N/A)	(N/A)	(N/A)
	2006-2010 (n=58)	18/58 (31.0 [19.5-44.5])	1/58 (1.7 [0.0-9.2])	18/58 (31 [19.5-44.5])	(N/A)	0/58 (0.0 [0.0-6.2*])
	2011-2015 (n=0)	(N/A)	(N/A)	(N/A)	(N/A)	(N/A)
	2016-2020 (n=0)	(N/A)	(N/A)	(N/A)	(N/A)	(N/A)
FSW	Before 2005 (n=20)	3/20 (15.0 [3.2-37.9])	1/20 (5.0 [0.1-24.9])	2/20 (10.0 [1.2-31.7])	3/20 (15.0 [3.2-37.9])	0/8 (0.0 [0.0-36.9])
	2006-2010 (n=90)	6/90 (6.7 [2.5-13.9])	3/90 (3.3 [0.7-9.4])	2/90 (2.2 [0.3-7.8])	1/90 (1.1 [0.0-6.0])	0/8 (0.0 [0.0-36.9])
	2011-2015 (n=10)	3/10 (30.0 [6.7-65.2])	2/10 (20 [2.5-55.6])	1/10 (10.0 [0.3-44.5])	0/10 (0.0 [0.0-30.8*])	(N/A)
	2016-2020 (n=26)	10/26 (38.5 [20.2-59.4])	10/26 (38.5 [20.2-59.4])	5/26 19.2 (6.6-39.4)	0/26 (0.0 [0.0-13.2*])	(N/A)
Children	Before 2005 (n=4)	0/4 (0.0 [0.0-60.2*])	0/4 (0.0 [0.0-60.2*])	0/4 (0.0 [0.0-60.2*])	0/3 (0.0 [0.0-70.6*])	(N/A)
	2006-2010 (n=0)	(N/A)	(N/A)	(N/A)	(N/A)	(N/A)
	2011-2015 (n=71)	31/71 (43.6 [31.9-56.0])	29/71 (40.8 [29.3-53.2])	15/71 (21.1 [12.3-32.4])	(N/A)	(N/A)
	2016-2020 (n=0)	(N/A)	(N/A)	(N/A)	(N/A)	(N/A)

*Abbreviations: MSM, men who have sex with men; PWID, people who inject drugs; FSW, female sex worker; HET, at-risk men and women who did not report sex work or male same-sex behaviour; NRTI, nucleoside reverse transcriptase inhibitors; NNRTI, non-nucleoside reverse transcriptase inhibitors; PI, protease inhibitors; INSTI, integrase strand transfer inhibitors. * One-sided, 97.5% confidence interval.*

Table S7. A summary of Dunn's post hoc test of multiple comparisons using rank sums indicating the temporal differences in nucleoside and non-nucleoside reverse transcriptase inhibitor mutations among different HIV-1 risk groups in Kenya.

Risk group category	Calender year (range)	Any HIVDR mutation		NNRTI		NRTI	
		Mean rank difference	P-value (adjusted)	Mean rank difference	P-value (adjusted)	Mean rank difference	P-value (adjusted)
**Overall (all risk groups combined)**	2006-2010 - before 2005	3.5	0.002	3.9	0.000	1.2	0.688
	2011-2015 - before 2005	7.2	0.000	8.1	0.000	3.3	0.003
	2016-2020 - before 2005	6.0	0.000	7.1	0.000	1.9	0.158
	2011-2015 - 2006-2010	5.9	0.000	6.5	0.000	3.2	0.004
	2016-2020 - 2006-2010	4.3	0.000	5.4	0.000	1.4	0.531
	2016-2020 - 2011-2015	0.8	1.000	1.4	0.449	-0.5	1.000
**HET**	2006-2010 - before 2005	3.5	0.001	4.3	0.000	1.1	0.832
	2011-2015 - before 2005	6.5	0.000	7.1	0.000	2.9	0.011
	2016-2020 - before 2005	0.0	1.000	0.5	1.000	-0.2	1.000
	2011-2015 - 2006-2010	4.6	0.000	4.6	0.000	2.7	0.022
	2016-2020 - 2006-2010	-1.1	0.849	-0.8	1.000	-0.5	1.000
	2016-2020 - 2011-2015	-2.2	0.092	-1.9	0.171	-1.1	0.770
**MSM**	2011-2015 - 2006-2010	0.4	0.984	0.4	1.000	0.6	0.803
	2016-2020 - 2006-2010	2.5	0.020	2.6	0.015	2.1	0.054
	2016-2020 - 2011-2015	1.4	0.258	1.5	0.211	0.9	0.548
**FSW**	2006-2010 - before 2005	-0.9	1.000	-0.2	1.000	-1.2	0.644
	2011-2015 - before 2005	1.1	0.842	1.2	0.650	0.0	1.000
	2016-2020 - before 2005	2.2	0.084	3.6	0.001	1.2	0.662
	2011-2015 - 2006-2010	2.0	0.154	1.6	0.332	0.9	1.000
	2016-2020 - 2006-2010	4.0	0.000	5.0	0.000	3.0	0.008
	2016-2020 - 2011-2015	0.6	1.000	1.6	0.340	1.0	0.983

Abbreviations: NNRTI, non-nucleoside reverse transcriptase inhibitor; NRTI, nucleoside reverse transcriptase inhibitor; MSM, men who have sex with men; PWID, people who inject drugs; FSW, female sex worker; HET, at-risk men and women who did not report sex work or male same-sex behaviour; Child, children.

Table S8. Estimated R_0 values of clusters having ≥3 sequences (n=5) with shared pre-treatment HIVDR mutations.

Estimated R_0 (95% HPD)									
**Cluster**	**Growth rate (95% HPD)**	**Where D=1 (95% HPD)**	**Where D=2 (95% HPD)**	**Where D=3 (95% HPD)**	**Where D=4 (95% HPD)**	**Where D=5 (95% HPD)**	**Where D=6 (95% HPD)**	**Where D=7 (95% HPD)**	**Where D=8 (95% HPD)**
#7.A1	-0.06 (-4.21-0.70)	0.94 (-3.21-1.70)	0.88 (-7.42- 2.40)	0.82 (-11.63- 3.10)	0.76(-15.84- 3.80)	0.70 (-20.05-4.50)	0.64 (-24.26-5.20)	0.58 (-28.47-5.90)	0.52 (-32.68-6.60)
#8.A1	0.10 (-6.08-10.28)	1.10 (-5.08- 11.28)	1.20 (-11.16- 21.56)	1.30 (-17.24-31.84)	1.40 (-23.32-42.12)	1.50 (-29.4-52.4)	1.60 (-35.48-62.68)	1.70 (-41.56-72.96)	1.80 (-47.64 -83.24)
#21.A1	0.38 (0.00-2.91)	1.38 (1.00-3.91)	1.76 (1.00 - 6.82)	2.14 (1.00 -9.73)	2.52 (1.00 -12.64)	2.90 (1.00 -15.55)	3.28 (1.00 -18.46)	3.66 (1.00 -21.37)	4.04 (1.00 -24.28)
#30.D	0.00 (0.00-4.37)	1.00 (1.00- 5.37)	1.00 (1- 9.74)	1.00 (1.00 -14.11)	1.00 (1.00 -18.48)	1.00 (1.00 -22.85)	1.00 (1.00 -27.22)	1.00 (1.00 -31.59)	1.00 (1.00 -35.96)
#10.A1	0.94 (0.00- 6.72)	1.94 (1.00-7.72)	2.88 (1- 14.44)	3.82 (1.00 -21.16)	4.76 (1-27.88)	5.70 (1-34.6)	6.64 (1.00 -41.32)	7.58 (1.00 -48.04)	8.52 (1.00 -54.76)

Bayesian dating was restricted to 5 clusters having ≥3 sequences and with the three most dominant pre-treatment HIVDR mutation per drug class i.e. NNRTI (K103NS, Y181CIV, and G190AES) and NRTI pre-treatment HIVDR mutations (M184VI and T215revs). The basic reproductive number (R_0, defined as the number of secondary infections that arise from a typical primary case in a completely susceptible population) per cluster was estimated based on the respective cluster growth rate (r) using the formula $R_0 = rD+1$ (where D represents the average duration of infectiousness for individuals – and in this study we assumed various values of D ranging from 1-8 years). Abbreviations: HPD (higher posterior density interval).
